# Supplementary material for: The interactome of intact mitochondria by cross-linking mass spectrometry provides evidence for coexisting respiratory supercomplexes
Source: Mol Cell Proteomics. 2017 Dec 8;17(2):216–32. doi: 10.1074/mcp.RA117.000470 (PMC5795388; doi:10.1074/mcp.RA117.000470)
Supplement: Supplemental Data [file supp_RA117.000470_133922_0_supp_23978_fzffwf.zip › spectra_annotation/mito_spectra_annotation/329-1-7-1-9-1.pdf]

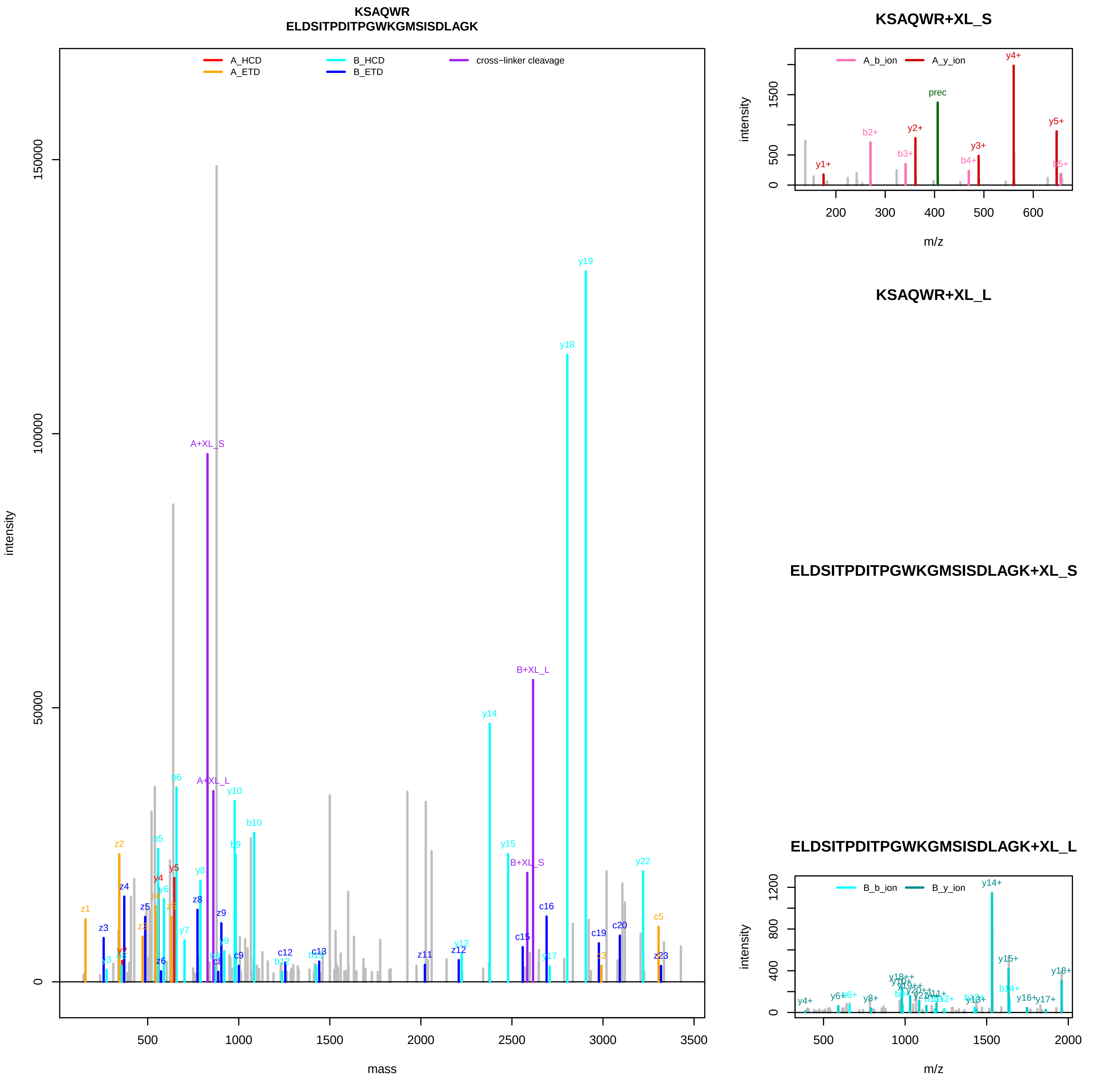
KSAQWR
ELDSITPDITPGWKGMSISDLAGK
A_HCD
A_ETD
B_HCD
B_ETD
cross−linker cleavage
intensity
mass
0
50000
100000
150000
500
1000
1500
2000
2500
3000
3500
A+XL_S
A+XL_L
B+XL_L
B+XL_S
y19
y18
y14
y15
y22
y10
b10
b6
b5
y8
z2
z1
z3
z4
z5
z8
z9
c9
c12
c13
z11
z12
c15
c16
c19
c20
z23
c5
y4
y5
y7
y12
y17
KSAQWR+XL_S
A_b_ion
A_y_ion
prec
y1+
b2+
y2+
b3+
y3+
b4+
y4+
y5+
m/z
200
300
400
500
600
1500
KSAQWR+XL_L
ELDSITPDITPGWKGMSISDLAGK+XL_S
ELDSITPDITPGWKGMSISDLAGK+XL_L
B_b_ion
B_y_ion
y14+
y15+
y18+
y4+
y6+
b6+
y8+
y13+
b14+
y16+
y17+
400
800
1200
2000
